# A quantitative analysis of 3D-cell distribution in regenerative muscle-skeletal system with synchrotron X-ray computed microtomography

**DOI:** 10.1038/s41598-018-32459-2

**Published:** 2018-09-20

**Authors:** Markéta Tesařová, Lucia Mancini, Andras Simon, Igor Adameyko, Markéta Kaucká, Ahmed Elewa, Gabriele Lanzafame, Yi Zhang, Dominika Kalasová, Bára Szarowská, Tomáš Zikmund, Marie Novotná, Jozef Kaiser

**Affiliations:** 1 Central European Institute of Technology, Brno University of Technology, Brno, Czech Republic; 2 Elettra-Sincrotrone Trieste S.C.p.A., Basovizza, Trieste Italy; 3 Department of Cellular and Molecular Biology, Karolinska Institutet, Solna, 171777 Stockholm Sweden; 4 Department of Physiology and Pharmacology, Karolinska Institutet, Solna 171777 Stockholm, Sweden; 5 Department of Molecular Neurosciences, Medical University Vienna, Vienna, Austria; 6 Department of Orthopaedics, Xiangya Hospital, Central South University, Changsha, Hunan Province China

**Keywords:** Cartilage development, Imaging techniques

## Abstract

One of the greatest enigmas of modern biology is how the geometry of muscular and skeletal structures are created and how their development is controlled during growth and regeneration. Scaling and shaping of vertebrate muscles and skeletal elements has always been enigmatic and required an advanced technical level in order to analyse the cell distribution in 3D. In this work, synchrotron X-ray computed microtomography (µCT) and chemical contrasting has been exploited for a quantitative analysis of the 3D-cell distribution in tissues of a developing salamander (*Pleurodeles waltl*) limb – a key model organism for vertebrate regeneration studies. We mapped the limb muscles, their size and shape as well as the number and density of cells within the extracellular matrix of the developing cartilage. By using tomographic approach, we explored the polarity of the cells in 3D, in relation to the structure of developing joints. We found that the polarity of chondrocytes correlates with the planes in joint surfaces and also changes along the length of the cartilaginous elements. Our approach generates data for the precise computer simulations of muscle-skeletal regeneration using cell dynamics models, which is necessary for the understanding how anisotropic growth results in the precise shapes of skeletal structures.

## Introduction

Several experimental techniques have recently been used for the visualization of cells in three dimensions (3D). These techniques usually use electromagnetic radiation with wavelengths of visible light or X-rays (wavelength 10^−6^–10^−10^ m). Mainly due to the shape and composition of the investigated samples, all imaging methods have their own advantages and limitations. The 3D biological structures, in their natural shape, may be thick and highly scattering, preventing e.g. light from penetrating them without significant distortion. Advanced light microscopy techniques can image thicker biological specimens at a high spatial resolution such as confocal microscopy, multiphoton microscopy, and optical coherence tomography^1^.

Confocal microscopy is considered to be one of the most convenient techniques for imaging cells in 3D. Moreover, this method has also been used specifically for the study of cell columns in the articular cartilage of rats^2^. Also, an *in vivo* study of the collagen matrix was performed by high-resolution fluorescence confocal microscopy^3^. However, *in vivo*, fluorescence imaging or the immunostaining of labelled specific cells^4^ have high requirements for the sample preparation. Moreover, fluorescence-based techniques are used to visualize a fluorescent marker that was targeted to the structure of interest. The auto-fluorescent properties of the cells can provide sufficient contrast to allow for the identification of the desired structures^1^. However, they cannot be used for all types of tissues, which can limit the utilization of this technique.

Additionally, imaging techniques based on the scattering of light are not suitable for imaging larger samples, e.g. the whole limbs of vertebrates. The penetration depth of confocal microscopy is limited to less than 100 µm^5,6^. Surface imaging microscopy has been compared to confocal microscopy imaging in order to visualize larger samples, such as whole embryos^7^. Despite the high spatial resolution for the whole body of an embryo, the method is destructive and highly demanding in terms of sample preparation.

A deeper sample penetration for 3D imaging can be achieved by multi-photon microscopy. This technique provides deep penetration mainly because of a scattered signal of photons which results from localized nonlinear signal generation. It can allow for optical sectioning in samples on a millimeter thickness scale, depending on the tissue type^8^. Such a thickness is comparable with the size of the skeletal elements of some embryonic vertebrates. However, this method cannot be applied to biological samples in general. The three-dimensional resolution of a two-photon excitation microscope is identical to that achieved in an ideal confocal microscope, i.e. hundreds of nanometers^9^.

Optical coherence tomography is a technique, which is capable of obtaining images from even thicker tissue samples^10^. On the other hand, the spatial resolution is lower (of the order of 5–10 µm)^11,12^, which is not sufficient for a quantitative analysis of single cell distribution in vertebrates.

A number of improvements have developed in X-ray-based methods for cellular imaging. X-rays can penetrate cells and thick tissues (from millimeter- to centimeter-sized samples) without the need for sectioning the sample and it is possible to generate quantitative 3D data with spatial resolution of up to several dozen nanometers on the selected regions of interest^13–15^. Soft X-ray microscopy or tomography and coherent diffractive imaging are techniques that examine even the subcellular structures. The soft X-ray microscopes usually use wavelengths in the so-called water window – the region of the spectrum between the K shell absorption edges of carbon and oxygen, which are the typical components for biological tissues. Carbon and nitrogen compounds absorb these X-rays more than water^12,16^. However, visualizing an entire organ or a developing limb would generate an inhibitive amount of data. The feasible approach must allow for the 3D imaging of a large field of view (millimeter scale) with the eventual possibility of zooming into areas of interest^17^.

X-ray computed microtomography (µCT) is a non-destructive imaging method that provides high spatial resolution (from micron to sub-micron scale) of 3D data for samples with the size ranging from sub-millimeter to several millimeters. The result is a map of the X-ray attenuation coefficient within the sample volume and, if certain experimental conditions are fulfilled, then also phase changes can be detected^18,19^. Recent developments of this method have significantly advanced biological imaging.

In synchrotron facilities, the small angular source size, the high intensity and the nearly-parallel geometry of the X-ray beam makes it possible to obtain not only a high spatial resolution on the macroscopic samples, but also to exploit the transverse coherence properties of the X-ray beam. This allows a very simple experimental approach to be used in the propagation-based phase-contrast imaging (PCI)^20,21^. By using synchrotron radiation, µCT measurements of the different regions of the large-scale objects (centimeter range) can be imaged at high spatial resolution (on the micron scale)^22–28^. Furthermore, the application of PCI techniques could allow different tissues with similar chemical compositions and radioscopic density, to be distinguished^29,30^.

Staining with heavy elements such as iodine, tungsten or osmium-based compounds^31,32^ also enhances the contrast within the various types of tissues using advanced µCT instruments (either laboratory or synchrotron-based). A PCI of biological samples can be employed even for unstained samples^33–35^. However, the combination of phase-contrast with the increasing X-ray absorption contrast by staining gives excellent image quality with better voxel resolution than absorption µCT setups^25,26^.

The patterning of skeletal elements during limb development in salamanders differs from other tetrapods^36^. Salamanders show an anterior or preaxial dominance in the order of formation and ossification of the zeugopodial and autopodial elements. Moreover, some proximal elements condense later than proximal ones^37^, which is definitely isolated from the familiar order of proximal to distal limb development in other tetrapods. In contrast with mammals, salamanders have a remarkable ability to regenerate their extraordinary range of body structures such as limbs, tails, retina, and spinal cord, along with some sections of the heart and brain^38^. Limb regeneration depends on the formation of a blastema, which is a pool of progenitor cells that arise after amputation. Blastema cells redifferentiate, proliferate and then restore the structured limbs^39^. The basic anatomy of the salamander joints, with the apposed, articular surfaces between the adjacent long bones, encapsulated by the connective tissues, is very similar to mammals^40–42^.

In many cases, the oriented cell dynamics and proliferation play an essential role in shaping and scaling, but the directional deposition of the matrix in the bone and cartilage is not very well understood. The majority of work so far has concentrated mainly on the analysis of the epithelial or migratory mesenchymal populations during limb or facial development, which has produced important knowledge on the asymmetric proliferation of mesenchyme and convergent extension processes in shape-making, in general^43–45^. However, the sculpting of precise and highly complex cartilaginous structures has not been recently focused upon except for several published works^46–50^. Despite attention to the cartilage shape, current works do not provide any comprehensive explanation for shape-making, but rather focuses on the importance of a multitude of factors for different aspects of cartilage and mesenchyme development with some general impact on shape.

Muscle is a tissue that lies immediately next to the developing cartilage tissue in the embryo and remains in close proximity to the cartilage template after birth^51,52^. On the one hand, the mechanical forces, induced by muscle contraction, seem to directly influence the morphogenesis regulation. Animal models, where muscle contractions have been removed or altered, and mouse mutants, without forming skeletal muscles, have led to the underdeveloped and misshapen skeletal elements^53^. Similar to humans, a short stature and scoliosis are common features in children with Duchenne Muscular Dystrophy^54^. Moreover, the immobilized muscle can also lead to joint structures loosening, such as a cavity, articular surfaces and patella^53^. On the other hand, muscle cells can release biochemical signals to regulate the cartilage gene expression indirectly^55^. It is still unknown how the precise, three-dimensional shape of skeletal elements is established, scaled up and what is the interaction in muscle and cartilage development. Finding a way to visualize and analyse cell distribution in 3D would be a great step forward to answering these biological issues. 3D morphological characterization at cellular resolution using synchrotron-generated X-rays has already been successfully demonstrated. Furthermore, the spatial distribution of single cells inside the articular cartilage has been proven on stained^56^ and unstained samples^57^. Other tissues, such as Purkinje cells in the brain, were quantified using automated cell counting^58^. However, previous approaches did not suffice in analysing cell polarization and the complex organization of multiple tissue types simultaneously with other 3D-analysis.

Here, we took advantage of the synchrotron-based X-ray μCT technique in combination with chemical contrasting in mapping the actual cells, their orientation and extracellular matrix distribution in 3D, during salamander (*Pleurodeles waltl*) limb development in the simultaneous analysis of cartilage and muscles. We decided to use the larval limb of a Spanish ribbed newt because it is a promising emerging model of limb regeneration and development^59^. The developing cartilages of *P*. *waltl* limbs are of the suitable size and are in the active growing and shaping phase. Our results demonstrated how the cell density and polarization within individual cartilaginous elements can be measured highlighting the localized accumulations of the extracellular matrix and changes in cell distribution and polarization during cartilage development. The method allowed for the mapping and 3D-reconstruction of several different tissue types at the same time, which is essential for understanding the development of a muscle-skeletal system. This study is a proof of the principle which shows the opportunity to use this type of analysis for exploring and modeling the development of skeletal structures with single-cell resolution.

## Results

### X-ray microtomography measurements

**Figure 1:**
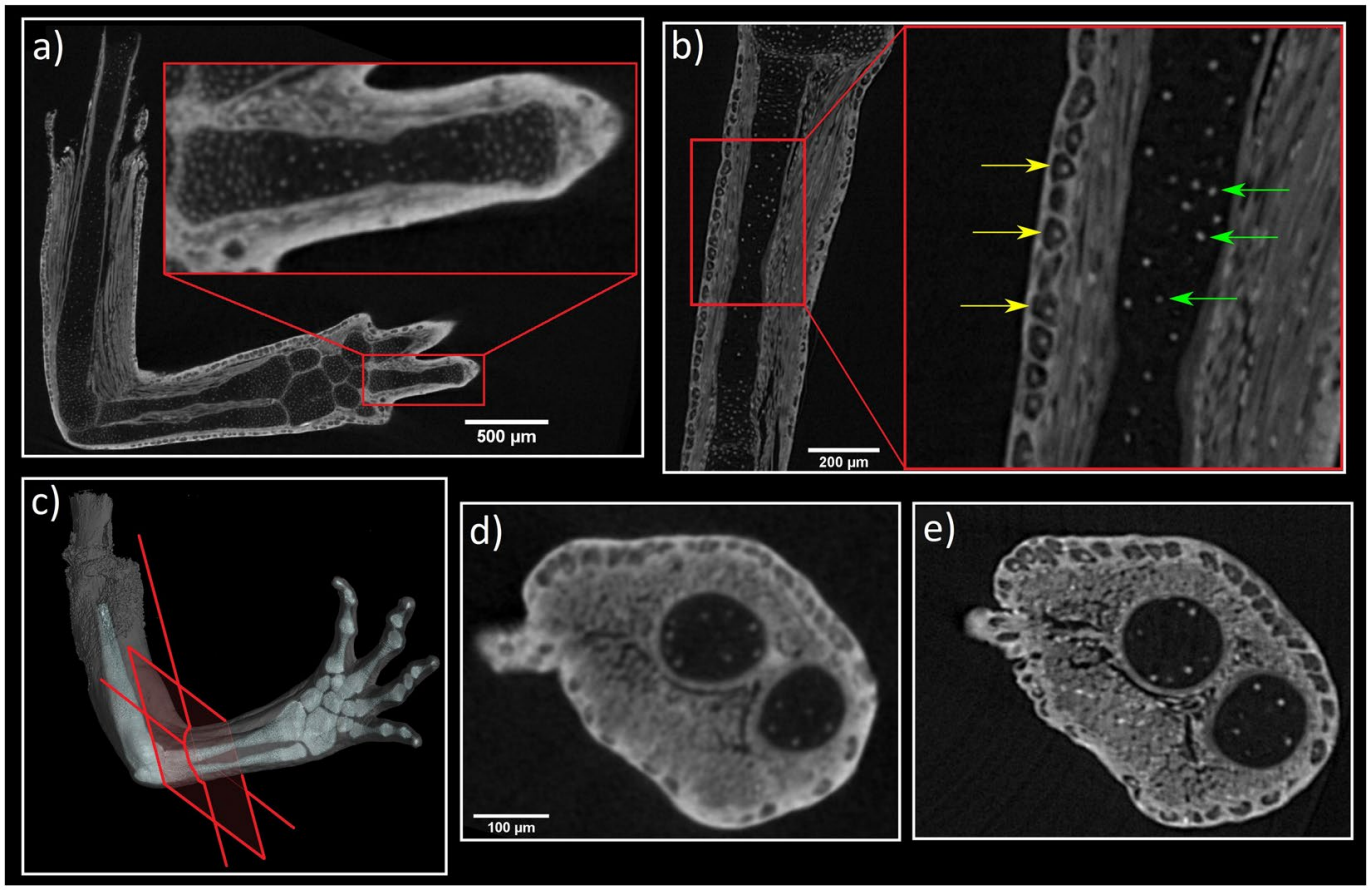
Comparison of the tomographic slices of a *P*. *waltl* forearm obtained by conventional and synchrotron µCT. Despite near-cellular resolution, the employed conventional µCT setup does not provide sufficient resolution for the automatic counting of cells with necessary accuracy. (**a**) Tomographic slice of the limb, the red area shows detail of one finger, (**b**) reconstructed slice obtained by phase-contrast µCT at the SYRMEP beamline of the Elettra synchrotron facility, yellow arrows show cells of skin epithelium, green arrows show nuclei of cartilage, (**c**) 3D visualization of a limb with red plane representing the same plane of interest of tomographic slices: (**d**) Data obtained by conventional µCT and (**e**) by synchrotron µCT.

The conventional X-ray µCT was exploited for overview experiments and the 3D visualization of developing *P*. *waltl* limbs stained with phosphotungstic acid (PTA). Here, we achieved an exceptional quality in chemical contrasting of the samples. However, the employed, conventional µCT instruments, did not provide sufficient resolution for a quantitative analysis and counting every single cell. Nevertheless, the resolution delivered by conventional µCT, gave basic information about qualitative cell distribution in the cartilage element (Fig. 1a,d). Though the information was only near-cellular, it was possible to detect some cell nuclei inside the limb.

The high photon flux, X-ray beam geometry and high spatial resolution (down to 1 µm) of synchrotron µCT achieved the cellular resolution for a quantitative analysis of cell distribution (see Fig. 1b,e). Moreover, the possibility to perform PCI experiments allowed the borders of cells in cartilaginous elements to be distinguished. A comparison of the same slice from conventional and synchrotron X-ray µCT is reported in Fig. 1. A video of raw tomographic slices obtained by synchrotron µCT measurement is in Supplementary Material 1.

### Data processing and analysis

Obtaining high-contrast tomographic data is the first step in 3D analysis. By chemical contrasting, the cartilage is significantly less stained in comparison to the surrounding tissue, which leads to easier discrimination and outline of cartilage in µCT data^48–50,60^. The reason is that PTA provides a strong X-ray contrast as it is attached to proteins such as collagens and fibrils^61,62^. Another important part is the data processing and analysis of the reconstructed tomographic volumes. Post-reconstruction data treatment requires segmentation of the investigated cartilage or bone elements by applying the appropriate algorithms for distinguishing every single cell in the desired area and then its quantitative analysis.

**Figure 2:**
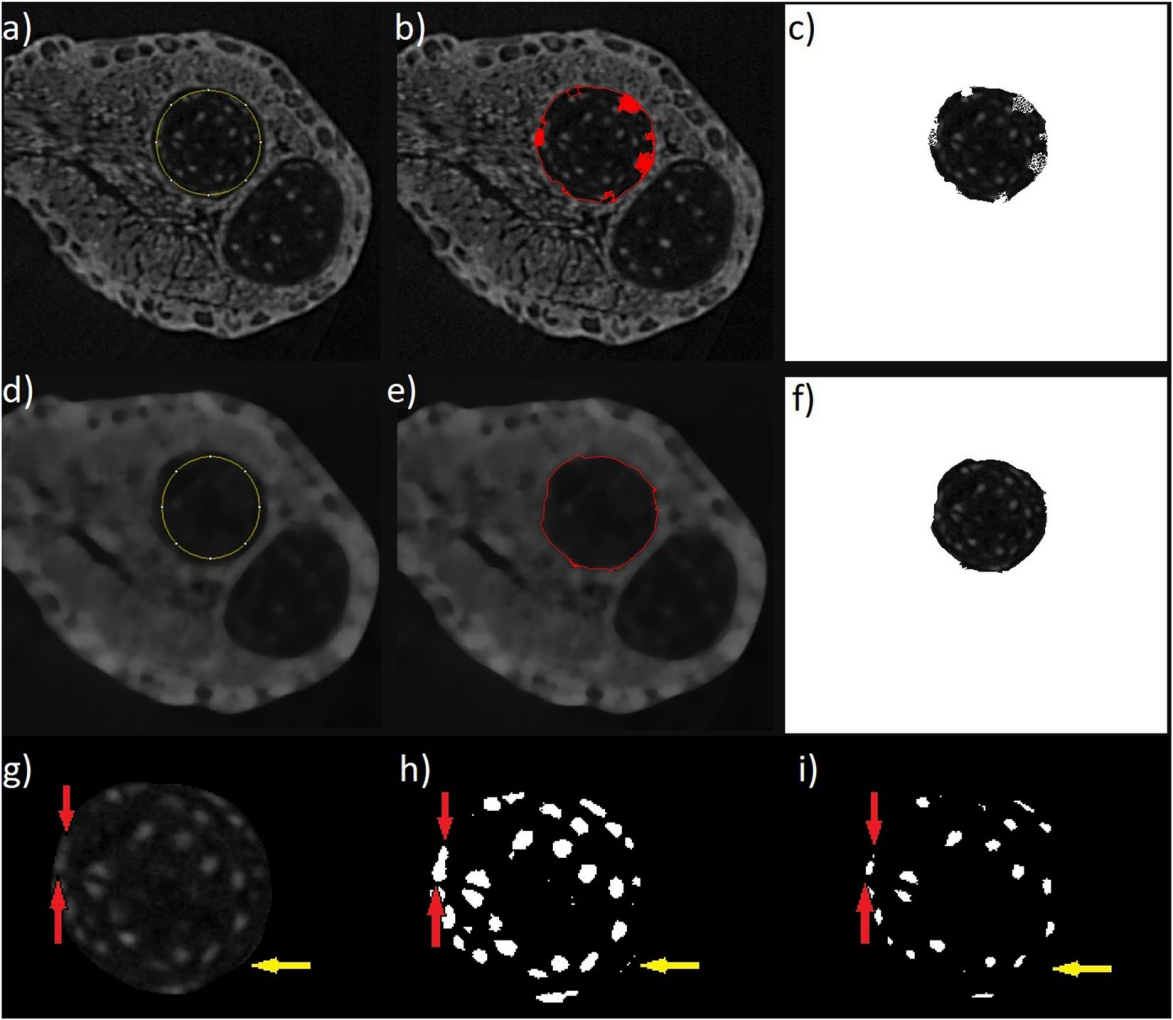
Automatic segmentation of skeletal elements performed by ABSnake^65,66^. (**a–c**) Shows the contour applied to non-filtered tomographic slices; however, rough borders caused errors. (**d–f**) Shows the contour applied to a filtered image. The mask was obtained without any errors and the shape of the resulting data appears almost perfect. (**g–i**) *Blob analysis* in the *Pore3D* software^67^. (**g**) One of the reconstructed slices used for the analysis, after the application of the 3D K-means clustering algorithm, (**h**) the results of the *blob analysis*, (**i**) the eroded segmented image. Red arrows indicate two nuclei of the cells that were connected within the *blob analysis*. The erosion of the binary image splits the blob into two parts. Yellow arrows show the light border of the cartilaginous element which should not be counted in the analysis. The erosion of the image also solved this problem.

First, the detection and separation of a cartilaginous element is needed with segmentation. Conventional segmentation algorithms using the region growing method^63^ are not suitable, due to the white spots inside the cartilage. These spots represent cell nuclei. Here, we applied the freeware image analysis software ImageJ^64^ with its plug-in ABSnake^65,66^. The next steps are to determine the starting contour for segmentation, the gradient threshold to be used in edge detection and the number of iterations of the segmentation cycles. Nevertheless, by applying a contour on the non-filtered tomographic slices, the iteration process does not converge to the border of the cartilage (Fig. 2a–c). This is due to the light spots (cell nuclei) in the cartilage body. Thus, the segmentation is not precise enough for the analysis. To avoid this problem, the contour is applied on the 3D median-filtered images. By filtering, the iteration procedure converges and the result of the process is the definition of the cartilage border. The final contour is smooth and perfectly copies the border of the cartilage body (Fig. 2d–f). The segmented data (cartilage element) can then be utilized for further analysis.

The next step for the quantification of cells was to count the cell nuclei represented by light spots in the image. For this purpose, the *Pore3D* software library^67^ developed at Elettra, was employed. The 3D K-means clustering algorithm was used to sort data into three classes. The K-means algorithm converts an input image into vectors of equal size and then by minimizing the sum of the squared distances from all points in a class to the class center^68^. This way, the binary images were obtained for the nuclei of cells, extracellular matrix and the background. The binary image for the class, representing the cell nuclei (binary large object – *blob*) is shown in Fig. 2h.

By comparing Fig. 2g,h, it is evident that some nuclei are connected into one *blob*. This example is shown by the red arrows in Fig. 2. Moreover, some border segments of the cartilage are miss-detected as *blobs* (yellow arrows in Fig. 2). To correct these analytical issues, the erosion of the 3D data is the next step. After this step, the data is well suited to determine the number of cells (Fig. 2i). To obtain the final number of cells, the binary data of cell nuclei after erosion (Fig. 2i) was implemented for further *blob analysis*^69^.

Basically, the limit of this type of analysis is determined by the resolution (spatial and contrast) of the system and by the size of the analysed structure. The spatial resolution of the detection system and the detectability of the given features of interest can be further influenced by working in phase-contrast mode^70^. In our 3D image processing and analysis, the objects considered as cells and included in the computations, were those with size larger than 3 pixels = 27 voxels (=27 μm^3^ per *blob*). The video of segmented data is shown in Supplementary Material 2.

### Biological results

**Figure 3:**
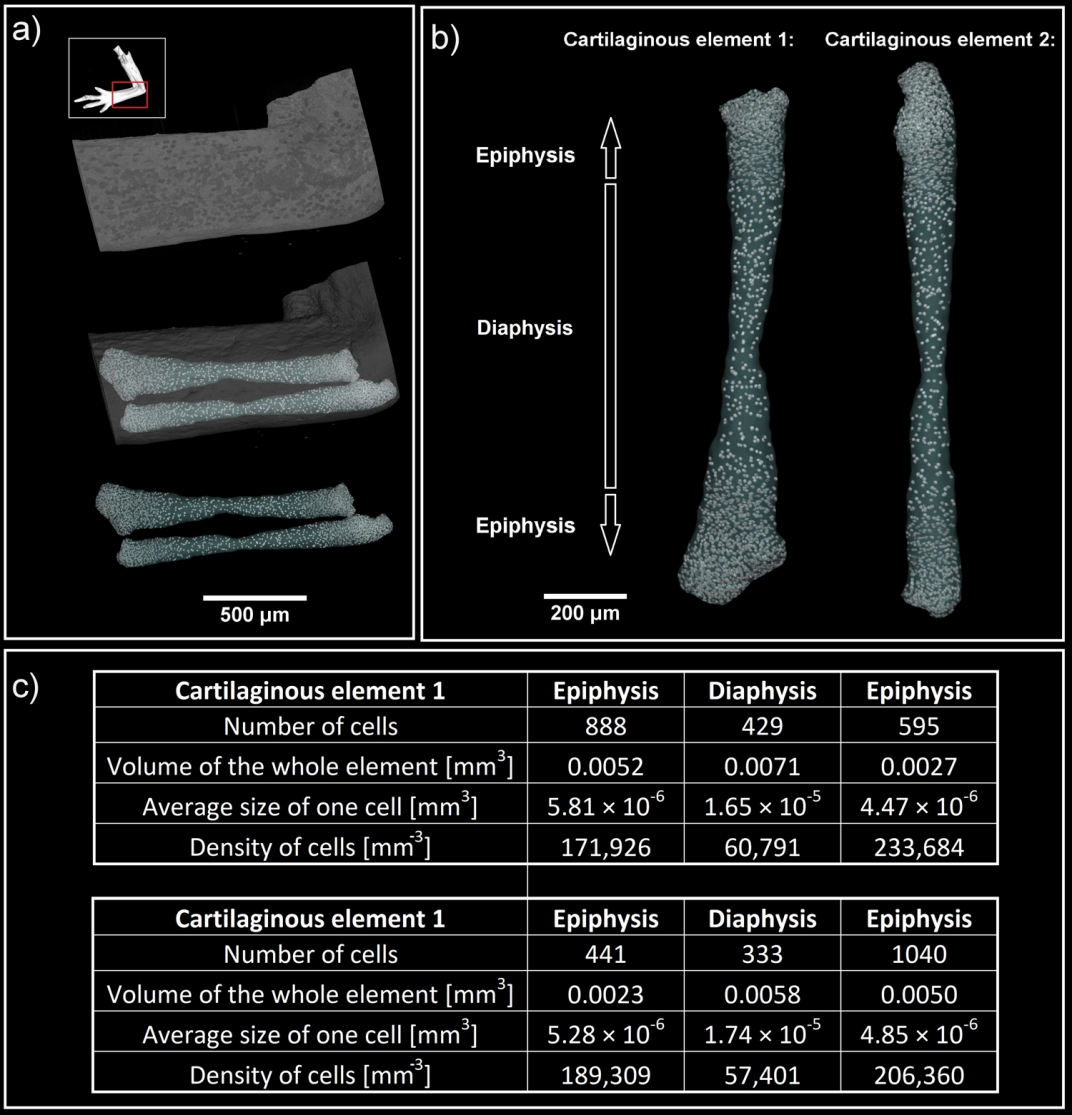
(**a**,**b**) 3D visualization of cartilaginous elements from a developing *P*. *waltl* limb forearm. White spots represent cell nuclei. c) Quantitative analysis allowed the determination of cell number, average size (given by ratio of volume of the whole element and number of cells), density and the volume of each cartilaginous element. The difference between diaphysis and epiphysis is evident: the density of cells in the diaphysis is half in comparison with the density in the epiphysis.

Our analysis of developing ulna and radius cartilages from a *P*. *waltl* limb demonstrated zonation that included a wide central region inhabited by the chondrocytes submerged in much larger amounts of extracellular matrix as compared to developing epiphyseal regions defined based on the instant changes of a cell density on a histogram. The cell number of these sparse chondrocytes in the central regions of both the ulna and the radius appeared low, which could reflect the reduction of cell number by decreased proliferation and acquisition of a hypertrophic stage. According to a classical model, epiphyseal chondrocytes continue proliferation longer than chondrocytes in a central region of a cartilaginous element and, consequently, show much higher density according to our data (Fig. 3).

**Figure 4:**
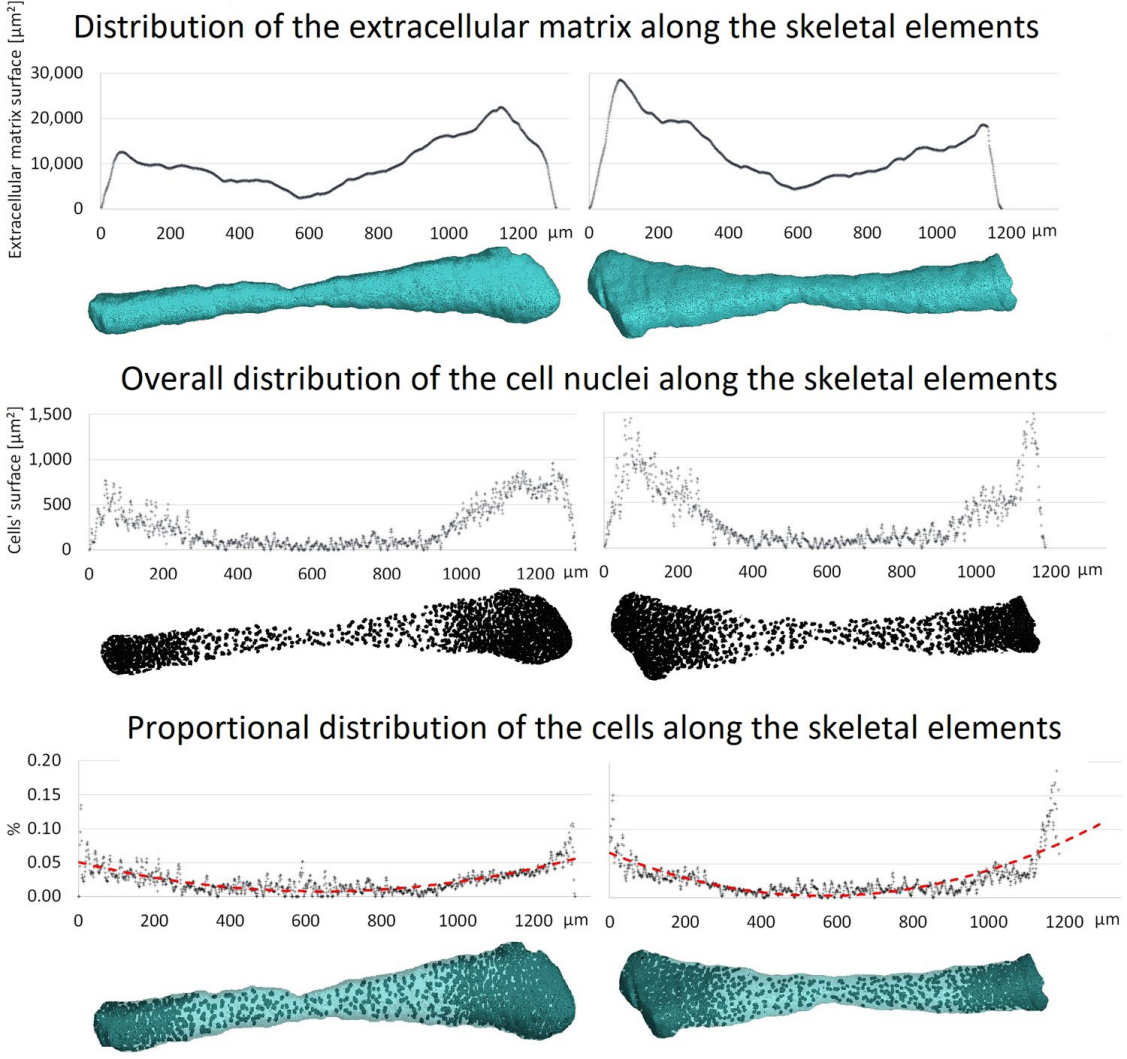
Distribution of extracellular matrix and cell nuclei along skeletal elements. Top: distribution of the area of extracellular matrix along the element. Middle: distribution of number of cells represented by the area of cell nuclei. Bottom: ratio between the area of cell nuclei and the area of extracellular matrix; the red-dotted line fits the data by a second order polynomial. The minimum of the parabola sits in the centre of the diaphyseal regions.

Further analysis showed more detailed distribution of cartilage along the growing skeletal element. For each slice, the area of the cell nuclei and area of the whole extracellular matrix were computed. Both structures showed uneven distribution in the distal direction. Interestingly, the ratio between cell nuclei and extracellular matrix areas changes (from approx. 0.1% to 0.01% - Fig. 4). The determined density of cells corresponds to the literature, which records estimated values for the cell density in cartilage between 30,000 and 110,000 cells mm^−3^ by confocal imaging^71^ or digital volumetric imaging by Jadin *et al*.^72^. However, the distribution in epiphyseal and diaphyseal regions has not been further discussed.

**Figure 5:**
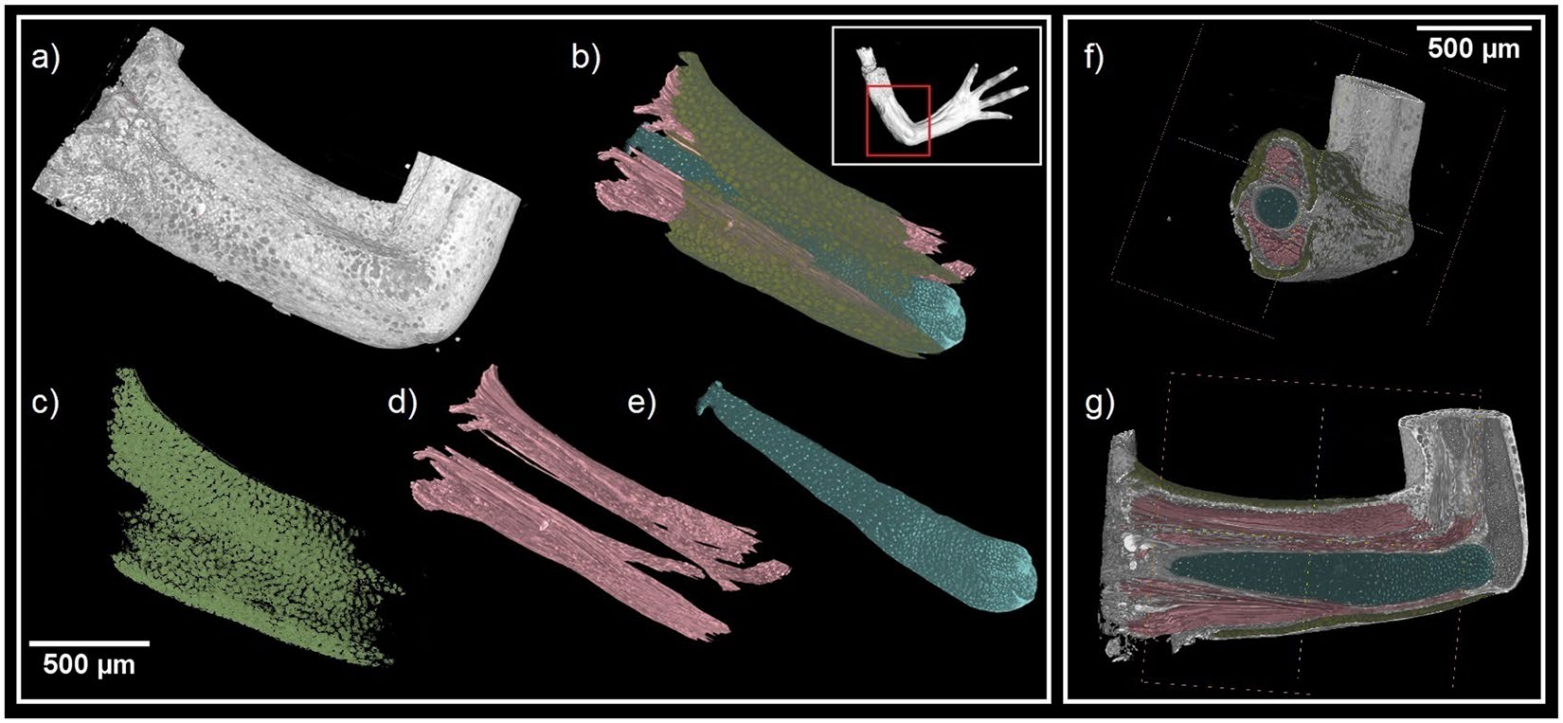
(**a**,**b**) 3D visualization of soft tissues showing other structures in the sample. (**c–e**) Sample segmentation showing cartilage (light blue), muscle fibers (red) and skin epithelium (yellow). (**f–g**) Clipping planes on the 3D model showing segmented structures inside the sample.

**Figure 6:**
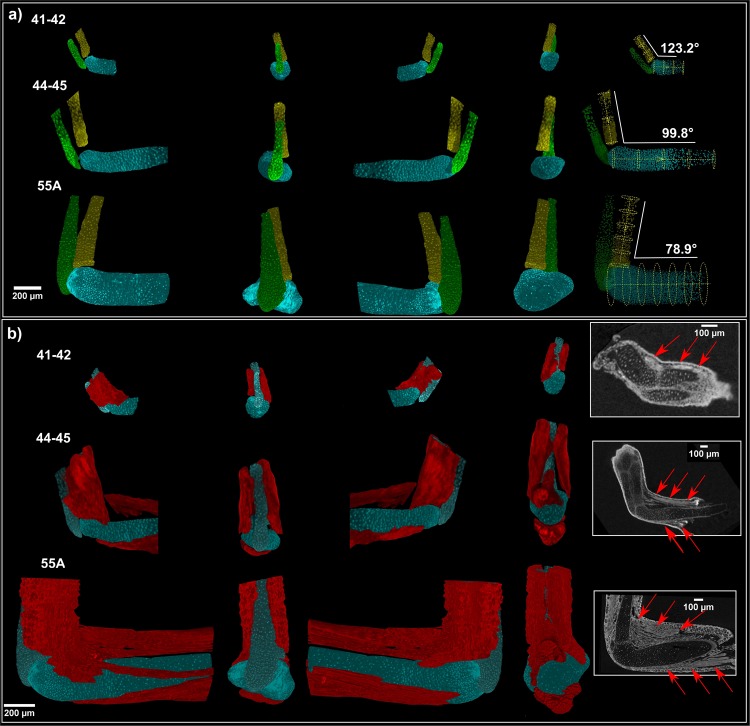
(**a**) Development of cartilage of the joint for three different developmental stages. The angle between *ulna* and *humerus* is decreasing with increasing developmental stage. (**b**) Visualization of joint simultaneously with developing muscles for three developmental stages. There is a correlation between decreasing angle and splitting of the muscles.

**Figure 7:**
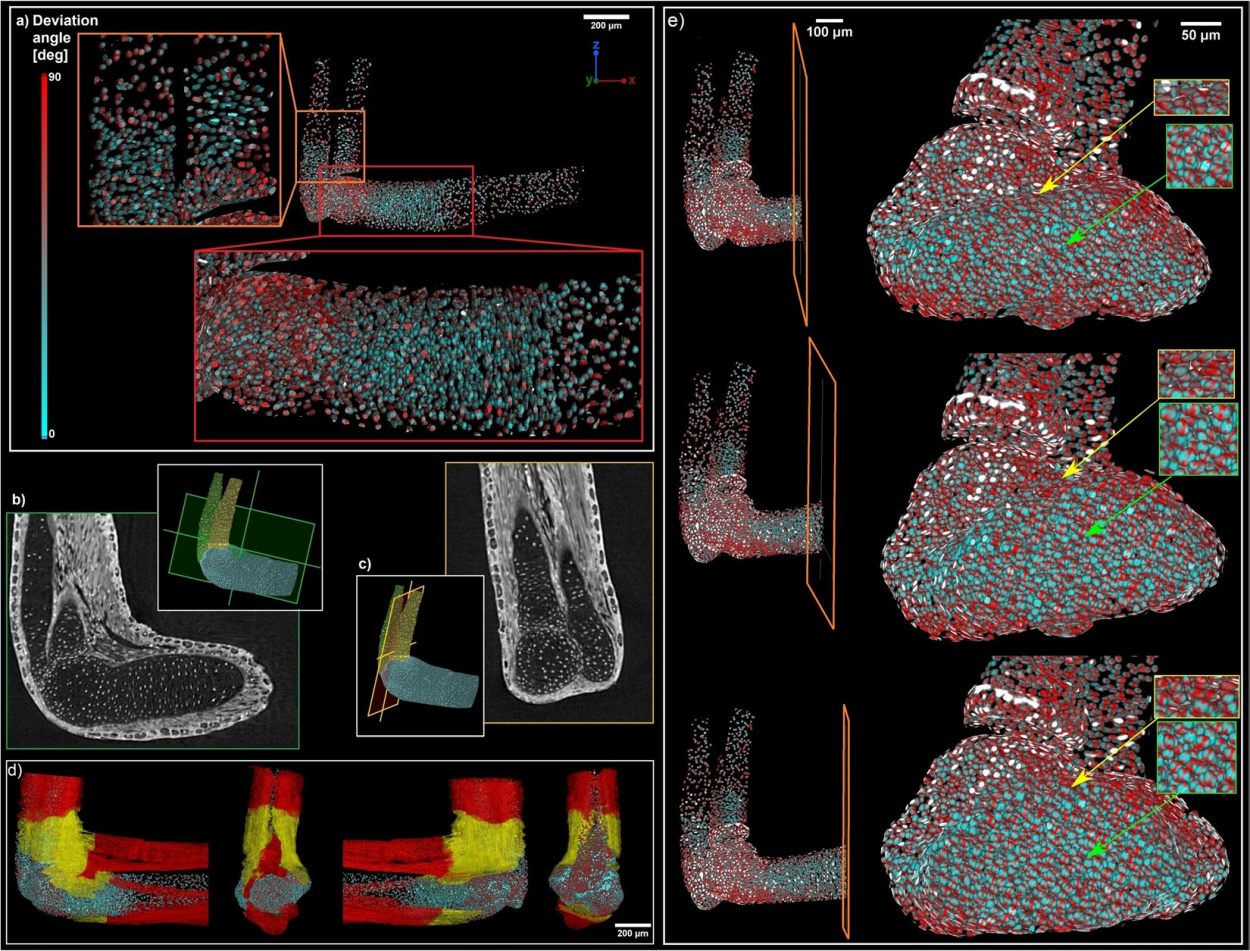
(**a**) Polarization of the cells inside cartilage. There are polarized zones between diaphysis and epiphysis for all cartilaginous elements: *ulna*, *radius* and *humerus*. Deviation angle 0° for *ulna* and *radius* was set to the plane *x*, *y* and for *humerus* was set to the plane *y*, *z;* (**b**,**c**) Corresponding tomographic slices verifying the computation of polarization; (**d**) Muscle attachment points in correlation with cell polarization; (**e**) Detail of orientation of the cells: Superficial chondrocytes near surface (yellow arrow) are aligned with the developing surface of the cartilage in the contrary of chondrocytes in the middle of the cartilage (green arrow).

A key advantage of synchrotron X-ray µCT measurements enhanced by PTA staining is the large variety of tissues that can be detected simultaneously. Information from all structures in a sample is included in one dataset obtained by a single measurement, which determines the position of each structure within the sample (Fig. 5, Supplementary Material 3). Efficient analysis can be done for a wide spectrum of tissues. This allows to reveal connections between muscles and cartilage development simultaneously. The muscles showed incremental growth in coordination with expanding cartilage. Also, we show the attachment points of muscles during the developmental process. The development of muscles then might guide cartilage and joint formation^73^. Three developmental stages were observed in muscle-skeletal point of view. At the youngest analyzed stage (41–42), only one small group of muscle around the elbow joint was found. However, different muscle group (*biceps brachii*, *triceps brachii*, *brachioradialis* and *flexor carpi radialis*) were recognized in the next two stages (Fig. 6b). Meanwhile, the elbow joint angle was decreasing with the development of muscle. Interestingly, the decreasing angle between *ulna* and *humerus* was observed with increasing developmental stages (Fig. 6a). Beyond question, the development of *biceps brachii* and *brachioradialis* may contribute to the decreasing elbow joint directly (the physical function of *biceps brachii* and *brachioradialis* is to bend the elbow joint). Another important finding is shape and position of the developing muscles. Surprisingly, there is no muscle splitting at the youngest analysed stage (41–42). The muscle splitting occurs later together with shaping of the cartilaginous joint. Furthermore, there is a correlation between the cell polarity inside the cartilage and splitting of the muscles (Fig. 7). The chondrocytes next to the muscles attachment points have a higher cell polarity. In another word, there is a correlation between the cell polarity inside cartilage and muscle attachment point. It is natural to speculate the mechanical force from muscle stretch may be delivered to attach point and then influence local chondrocytes polarity (Fig. 7d). It gives rise to a thought that a hitherto unknown mechanism controls the location and the density of chondrocytes together with cell polarity and muscle attachment points.

Our visualizations provided detailed information about developing joint surfaces at cellular resolution in 3D in the regenerative salamander limb. Development of surface geometry correlated in time with the formation of attached striated muscles. Orientation of chondrocytes in the developing joint, measured for the first time in the entire 3D volume by using X-ray computed tomography, correlated with the changing curvatures of joint surfaces. More generally, the resolution and differential contrast were sufficient to map the orientation of all chondrocytes within the cartilage, which provided important foundation for future inference of the oriented cell behaviour during cartilage shaping. Our results demonstrated that the predominant orientation of chondrocytes in epiphyseal regions was different from rather central regions of the cartilage, where the cell density appeared low. In addition to this, superficial chondrocytes in epiphyseal regions were aligned with the developing surface of the cartilage (Fig. 7e).

Taken together, our biological results show that development of cartilage geometry (including forming joints) correlates in time with developing skeletal muscles, which may influence the orientation of chondrocytes in epiphyseal regions, and through this, and in accordance with previously published data^73^, regulate morphogenesis and fine shaping of specific regions in cartilage.

## Discussion and Conclusion

In this study, we have demonstrated a novel, technical approach allowing for the quantitative analysis of polarization and 3D cell distribution inside the whole developing muscle-cartilaginous units from a regenerative animal model. This approach is based on the chemical contrasting of samples with PTA, followed by a high-resolution X-ray microtomography (μCT) measurement and the subsequent 3D data-processing and analysis. The polarization of the cell is determined by analysing the shape of cells in the matrix or the shape of their nuclei within other soft tissues. The best results, suitable for the quantitative analysis, with a single cell resolution and qualified for the computer simulations, were achieved by the phase-contrast synchrotron X-ray μCT analyses performed at the SYRMEP beamline of the Elettra facility. On the other hand, by using a conventional X-ray µCT instrument, the resolution turned out to be near-cellular, whereas the quantitative analysis faced a number of additional problems. Nevertheless, the data obtained by the conventional setup delivered important complementary information and allowed the visualization of many well-resolved internal structures.

Unexpectedly, our biological results show that the orientation of cells in the cartilage changes with cell density and position along the cartilaginous element and also in relation to the general position of the muscle attachment points and forming joint surfaces. The polarity of a cell may reflect so-called oriented cell behaviour, which might include oriented cell divisions, cell migration or other asymmetric processes important for shaping the structure^74^. Geometrical signs of cell polarisation in the cartilage, obtained from tomographic data, suggest the potential role of mechanical forces in cartilage geometry formation via the control of the chondrocyte cell orientation within the extracellular matrix. The polarity of chondrocytes and perichondrial cells in the attachment point areas may result from tension in the developing muscles, which we mapped in terms of geometry and volume. Previous findings already established the role of the developing muscles in cartilage shaping^73^. Brunt and co-authors^73^ demonstrated that muscles are important for the morphogenesis of joints by using zebrafish as a model system together with computer simulations. The authors of the study concluded that the mechanical strain created by the muscles, influences cell orientation in the developing jaw joint of a fish^73^, which goes along with our findings in the regenerative model system, where we provide 3D information of cell orientation suitable for further 3D-modeling efforts. On the other hand, according to our data, the muscles also expand in the direction following the attachment points that change their relative position in the growing cartilage with time.

In general, the obtained data, including the number of cells in 3D volume, their density, polarization, zonal distribution and the total volume of the skeletal elements, are important to understand growth, shaping, scaling and the regeneration of muscle and cartilaginous structures in the developing and regenerating vertebrates. Here, we have delivered proof of the principle study to show the possibility of visualizing and counting the individual chondrocytes in the cartilage of a vertebrate model system, suitable for both the developmental biology and the regenerative research. This approach can provide precise information about the incremental growth of a structure in terms of changes in cell polarity, cell numbers and simultaneously with the transforming shape. We envision that in the near future conventional laboratory µCT setups will gain similar imaging capabilities to the synchrotron beamlines, opening new possibilities of studying biological structures in 3D with single-cell resolution. Hereby, the use of such conventional µCT measurements in combination with open-source software for 3D image analysis, will enable new opportunities for the community of biologists and biomedical specialists, investigating the development and regeneration of skeletal and non-skeletal tissues.

## Methods

### Sample preparation

The *Pleurodeles waltl* (Spanish ribbed newt) colony was established from fertilized eggs produced in a laboratory colony located in Madrid, Spain. Animals originating from a wild population in the Doñana National Park (Spain) were obtained for research purposes by Agustin Gonzalez. The animals, used to prove the concept study were 4th/5th generation, developmental stage 41–42, 44–45 and 55 A (approximate length of the whole larva was 3.7 cm). Staging of the larva was performed according to Joven *et al*.^75^. The frontal amputated limbs were briefly washed in PBS and fixed in freshly mixed 4% PFA for 12 hours at +4 degrees. A contrasting of the developing limbs was performed as follows: the samples were dehydrated in increasing ethanol grade (30%, 50%, 70%, 90%) at room temperature, 2 hours for each step, using a slow rotation of the samples. The samples were transferred into a solution of 0.7% PTA (phosphotungstic acid) in 90% methanol and incubated at +4 degrees for 5 days using slow rotation and the PTA-contrasting solution was changed after every 24 hours for a fresh one. The samples were washed with 90% methanol overnight at +4 degrees and then rehydrated in a decreasing ethanol grade (90%, 70%, 50%, 30%) at room temperature, 2 hours for each step and slow rotation.

### Use of experimental animals

All procedures on newts were approved by local ethics committee (Stockholms Djurförsöksetiska Nämnd) and were performed in accordance with national regulations issued by the Swedish Board of Agriculture.

### Conventional X-ray µCT measurements

The contrast of the stained samples was checked by a conventional X-ray µCT. Following that, the developing limbs were embedded in 1.0% of agarose gel and placed in polypropylene tubes to avoid movement artefacts during tomography scanning. A polypropylene tube was fixed on a plastic rod by a silicone gun. The rod, containing the sample, was put in the centre of the rotation stage axis. A μCT scanning was performed using the laboratory system GE Phoenix v|tome|x L 240 (GE Sensing & Inspection Technologies GmbH, Germany) with a 180 kV/15 W maximum power nanofocus X-ray tube and a high contrast, flat panel detector DXR250 with 2048 × 2048 px2 and a pixel size of 200 × 200 μm^2^. Exposure time was 900 ms in each of the 2200 projections acquired over a total angle of 360°. The utilized acceleration voltage and the current of the X-ray tube were 60 kV and 200 μA, respectively. The beam was filtered by a 0.2 mm-thick aluminium filter to reduce beam-hardening artefacts. The tomographic reconstruction was done using the software GE phoenix datos|x 2.0 (GE Sensing & Inspection Technologies GmbH, Germany) with an isotropic voxel size of 2.5 μm.

### Synchrotron X-ray µCT measurements

Phase-contrast synchrotron X-ray µCT measurements were performed at the SYRMEP beamline of the Italian synchrotron radiation facility Elettra with the white beam mode. The X-ray spectrum of the beam was filtered with 1.5 mm of silicon and 0.025 mm of molybdenum. The sample-detector distance was set at 100 mm. The experiments were conducted with an isotropic voxel size of 1.05 μm. The exposure time per projection was 1.0 s with 1000 projections acquired over a total scan angle of 180°. Thus, the total scanning time was about 17 minutes. The tomographic slices were reconstructed using the SYRMEP Tomo Project (STP) software developed at Elettra^76^.

### Data processing and analysis

Reconstructed slices were further analysed using the freeware ImageJ^62^ and the *Pore3D* software library^65,67^. Firstly, the segmentation of cartilaginous elements was carried out using the plugin ABSnake^63,64^ with a gradient threshold of 30 and a setting of 50 iterations. This plugin was applied to the dataset filtered by a Median 3D filter with a radius of 10 in all three dimensions. The final cartilaginous element (i.e. developing *P*. *waltl* forearm) was obtained in ImageJ by using the segmented slices as a transparent-zero mask on the original, non-filtered dataset. Further analyses were carried out by using *Pore3D*. To separate the background, the extracellular matrix and the bright cell nucleus, a 3D K-means clustering algorithm was applied to divide the data into three classes. The binary image of the class represented by the cell nuclei was consequently processed by the *erosion* and the *blob analysis* modules of *Pore3D*, which allowed for the determination of the number of *blobs*, i.e. the number of cells. Cell polarization was determined using the software, VGStudio Max 3.1, with its module *Fiber orientation analysis*. The different tissues such as the skin epithelium and muscles were segmented semi-automatically in combination with the software Avizo and VGStudio Max 2.2 according to Tesarova *et al*.^60^.

## Electronic supplementary material

Supplementary information

Supplementary material 1

Supplementary material 2

Supplementary material 3

## Data Availability

The datasets generated and/or analysed during the current study are available in the Image Data Resource repository.
