# Author Correction: A quantitative analysis of 3D-cell distribution in regenerative muscle-skeletal system with synchrotron X-ray computed microtomography

**DOI:** 10.1038/s41598-020-59021-3

**Published:** 2020-02-05

**Authors:** Markéta Tesařová, Lucia Mancini, Andras Simon, Igor Adameyko, Markéta Kaucká, Ahmed Elewa, Gabriele Lanzafame, Yi Zhang, Dominika Kalasová, Bára Szarowská, Tomáš Zikmund, Marie Novotná, Jozef Kaiser

**Affiliations:** 1 Central European Institute of Technology, Brno University of Technology, Brno, Czech Republic; 2 Elettra-Sincrotrone Trieste S.C.p.A., Basovizza, Trieste Italy; 3 Department of Cellular and Molecular Biology, Karolinska Institutet, Solna, 171777 Stockholm Sweden; 4 Department of Physiology and Pharmacology, Karolinska Institutet, Solna, 171777 Stockholm Sweden; 5 Department of Molecular Neurosciences, Medical University Vienna, Vienna, Austria; 6 Department of Orthopaedics, Xiangya Hospital, Central South University, Changsha, Hunan Province China

Correction to: *Scientific Reports* 10.1038/s41598-018-32459-2, published online 20 September 2018

The original version of this Article contained an error in the title of the paper, where the word “regenerative” was incorrectly given as “regeneration”.

Additionally, in the Results section under subheading ‘Biological results’,

“The muscles showed incremental growth during the regeneration process in coordination with expanding cartilage and also appeared with their corresponding attachment points at different timing.”

now reads:

“The muscles showed incremental growth in coordination with expanding cartilage. Also, we show the attachment points of muscles during the developmental process.”

“Our visualizations provided detailed information about developing joint surfaces at cellular resolution in 3D in the regenerating salamander limb.”

now reads:

“Our visualizations provided detailed information about developing joint surfaces at cellular resolution in 3D in the regenerative salamander limb.”

These errors have now been corrected in the PDF and HTML versions of the Article and in the accompanying Supplementary Information file.

