# Supplementary material for: A quantitative analysis of 3D-cell distribution in regenerative muscle-skeletal system with synchrotron X-ray computed microtomography
Source: Sci Rep. 2018 Sep 20;8:14145. doi: 10.1038/s41598-018-32459-2 (PMC6148031; doi:10.1038/s41598-018-32459-2)
Supplement: Supplementary file 1 — Supplementary information [file 41598_2018_32459_MOESM1_ESM.docx]

**A quantitative analysis of 3D-cell distribution in regenerative muscle-skeletal system with synchrotron X-ray computed microtomography**

Markéta Tesařová^1^, Lucia Mancini^2^, Andras Simon^3^, Igor Adameyko^4,5^, Markéta Kaucká^4,5^, Ahmed Elewa^3^, Gabriele Lanzafame^2^, Yi Zhang^4,6^, Dominika Kalasová^1^, Bára Szarowská^4^, Tomáš Zikmund^1^, Marie Novotná^1^ and Jozef Kaiser^1*^

^1^Central European Institute of Technology, Brno University of Technology, Brno, Czech Republic

^2^Elettra-Sincrotrone Trieste S.C.p.A., Basovizza, Trieste, Italy

^3^Department of Cellular and Molecular Biology and ^4^Department of Physiology and Pharmacology, Karolinska Institutet, Solna 171777, Stockholm, Sweden

^5^Department of Molecular Neurosciences, Medical University Vienna, Vienna, Austria

^6^Department of Orthopaedics, Xiangya Hospital, Central South University, Changsha, Hunan Province, China.

*Corresponding author: [kaiser@fme.vutbr.cz](mailto:kaiser@fme.vutbr.cz)

**Supplementary information**

Supplementary material 1 – Video of raw CT slices of a salamander limb 55A.

Supplementary material 2 – Video of segmented slices of one cartilaginous element.

Supplementary material 3 – Video of 3D visualization of a salamander limb 55A showing a variety of soft tissues that can be visualized simultaneously: cartilage (light blue), muscle fibres (red) and skin epithelium (yellow).
